# Developments in respiratory self-management interventions over the last two decades

**DOI:** 10.1177/14799731231221819

**Published:** 2023-12-21

**Authors:** Tanja W Effing

**Affiliations:** 1 College of Medicine and Public Health, 1065 Flinders University of South Australia, Bedford Park, South Australia, Australia; 2 School of Psychology, Faculty of Health and Medical Sciences, 1066 University of Adelaide, Adelaide, South Australia, Australia

**Keywords:** Self-management, asthma, chronic obstructive pulmonary disease, effectiveness, adherence, tailoring, implementation

## Abstract

This paper describes developments in the fields of asthma and COPD self-management interventions (SMIs) over the last two decades and discusses future directions. Evidence around SMIs has exponentially grown. Efficacy on group level is convincing and both asthma and COPD SMIs are currently recommended by respiratory guidelines. Core components of asthma SMIs are defined as education, action plans, and regular review, with some discussion about self-monitoring. Exacerbation action plans are defined as an integral part of COPD management. Patient’s adherence to SMI’s is however inadequate and significantly reducing the intervention’s impact. Adherence could be improved by tailoring of SMIs to patients’ needs, health beliefs, and capabilities; the use of shared decision making; and optimising the communication between patients and health care providers. Due to the COVID-19 pandemic, digital health innovations have rapidly been introduced and expanded. Digital technology use may increase efficiency, flexibility, and efficacy of SMIs. Furthermore, artificial intelligence can be used to e.g., predict exacerbations in action plans. Research around digital health innovations to ensure evidence-based practice is of utmost importance. Current implementation of respiratory SMIs is not satisfactory. Implementation research should be used to generate further insights, with cost-effectiveness, policy (makers), and funding being significant determinants.

## Introduction

With the rising burden of chronic diseases, there is worldwide interest in how to better and more efficiently manage patients.^1^ Over the last two decades, asthma and COPD self-management interventions (SMIs) have played an important role in supporting people to optimise their self-management health behaviour on a day-to-day basis.^2^

The first asthma self-management paper was published at the end of the seventies.^3^ Knowledge that was gained in the asthma field was initially used to set up COPD SMIs.^4^ However, over the last 20 years, the fields of asthma and COPD self-management have each made significant steps forward, while mostly focusing on developing their own disease specific SMIs.^5,6^ Both asthma and COPD self-management are now recognised by the respiratory community and recommended by the guidelines.^7,8^

This overview paper describes the developments in the fields of asthma and COPD SMIs over the last two decades by discussing subsequently: definitions, body of evidence, action plans, patient adherence, the use of theories, tailoring of patient-centred approaches (including digital technology), and the implementation of SMIs. While reviewing developments over the past, potential future approaches are also discussed.

## Definitions of self-management interventions

One of the first to use the term self-management in the respiratory field were Creer et al. in 1978.^3^ They stressed the importance of self-management behaviour and indicated that children’s ability to manage their asthma needed to improve before they could return home after being admitted to an asthma facility.^3^ In 2003, Lorig et al. published a highly respected and frequently cited paper defining six important general self-management skills: problem solving, decision making, resource utilization, the formation of a patient-provider partnership, action planning, and self-tailoring.^2^

The first Cochrane reviews regarding asthma self-management education in adults were published more than two decades ago ^9,10^ and did not include definitions of SMIs and/or self-management behaviour. The core intervention components detected in the review of Gibson et al^9^ (education, self-monitoring, medical review, and a written action plan) have however been integrated in multiple asthma self-management definitions, including the definition of ‘guided self-management education’ in the latest Global Initiative for Asthma Strategy (GINA) (Table 1).^7^ It is notable that even in relatively recent asthma reviews,^5,11^ definitions of asthma-specific SMIs are frequently absent, instead authors choose to include a general SMI definition and/or a definition of asthma-specific self-management behaviour (Table 1).

**Table 1.** Definitions of a ‘self-management intervention’ or ‘self-management behaviour’ in some frequently cited publications in asthma and COPD.

Asthma	
BTS guideline 2016^103^ Harris et al. 2019^11^ Pinnock et al. 2017^5^	**Self-management behaviour:** Self-management are the tasks that individuals must undertake to live with chronic conditions, including have the confidence to deal with medical management, role management and emotional management of their conditions.
Global initiative for asthma strategy (GINA) 2023^7^	**Self-management intervention:** Guided self-management education includes monitoring of symptoms and/or lung function, a written action plan, and regular review by a health professional.
COPD	
Bourbeau et al. 2003^12^	**Self-management intervention:** Self-management is a term applied to any formalized patient education program aimed at teaching skills needed to carry out medical regiments specific to the disease, guide health behaviour change, and provide emotional support for patients to control their disease and live functional lives.
Effing et al. 2016^14^ GOLD guidelines 2023^8^	**Self-management intervention:** A COPD self-management intervention is structured but personalized and often multi-component, with goals of motivating, engaging and supporting the patients to positively adapt their health behaviour(s) and develop skills to better manage their disease. The ultimate goals of self-management are: (a) optimizing and preserving physical health; (b) reducing symptoms and functional impairments in daily life and increasing emotional well-being, social well-being and quality of life; and (c) establishing effective alliances with healthcare professionals, family, friends and community. The process requires iterative interactions between patients and healthcare professionals who are competent in delivering self-management interventions. These patient-centred interactions focus on (1): identifying needs, health beliefs and enhancing intrinsic motivations (2): eliciting personalized goals (3): formulating appropriate strategies (e.g. exacerbation management) to achieve these goals; and if required (4): evaluating and re-adjusting strategies. Behaviour change techniques are used to elicit patient motivation, confidence and competence. Literacy sensitive approaches are used to enhance comprehensibility.

Abbreviations: COPD: Chronic Obstructive Pulmonary Disease; BTS: British Thoracic Society; GOLD: Global Initiative for chronic Obstructive Lung Diseasse.

In 2003, a frequently cited COPD-specific SMI definition was published by Bourbeau et al.^12^ which was predominantly centred around education (Table 1). Between 2003 and 2016, most definitions of COPD SMIs involved slight alterations of this definition. As the term ‘education’ is often only limited to transmission of knowledge and sole education is not likely to lead to change of behaviour, these definitions were not considered as ideal.^13^ In 2016, a COPD expert group reached consensus regarding a conceptual definition of a COPD SMI, clarifying the boundaries of what should and should not be considered as a COPD SMI^14^ (see Table 1). Operationalisation of the conceptual definition of a self-management intervention would be desirable but has so far not been achieved.

Irrespective of the concerns of using the term ‘education’,^13,14^ the term is still commonly used, not only in the field of asthma but also in the field of COPD and is featured as so, in the most recent GINA and Global Initiative for Chronic Obstructive Lung Disease (GOLD) guidelines.^7^ In addition, the term ‘supported SMI’ is frequently used in the asthma self-management field.^5^ Whether to add ‘supported’ to ‘SMI’, was a point of discussion in the COPD SMI definition Delphi study.^14^ As ‘support’ was considered as being intrinsic to a SMI by most of the study participants, it was decided to leave ‘supported’ out.^14^ Finally, in COPD literature, the term ‘self-management’ has been frequently used to refer to both the intervention and the behaviour over the last 20 years. Recently, the term is however more often accurately used to refer only to self-management behaviour, not the intervention.^14^

**Figure 1.**
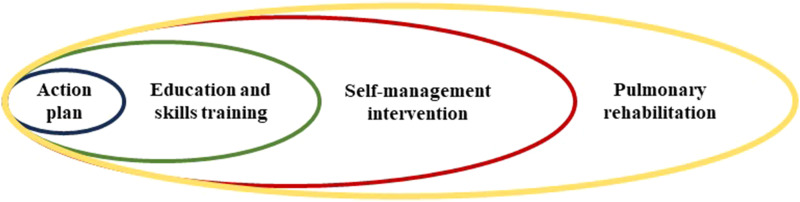
An adopted visualisation of Wagg (2012)^15^ showing how a self-management intervention relates to pulmonary rehabilitation, education and skills training, and an action plan.

Over the last decade, there has been discussion of how to position SMIs in the field of respiratory medicine, especially with regard to pulmonary rehabilitation (PR). In 2012, Wagg^15^ nicely visualised how PR, self-management, education, and action plans related to each other (adaptation of Wagg's visualisation: Figure 1). Nowadays it is clear that PR programmes should ideally integrate self-management approaches to achieve improved health behaviours and behavioural change,^8^ SMIs are however considered as self-contained treatment options^7,8^ that need to be integrated within the routine of healthcare services.^16^

## Growing body of evidence

**Figure 2.**
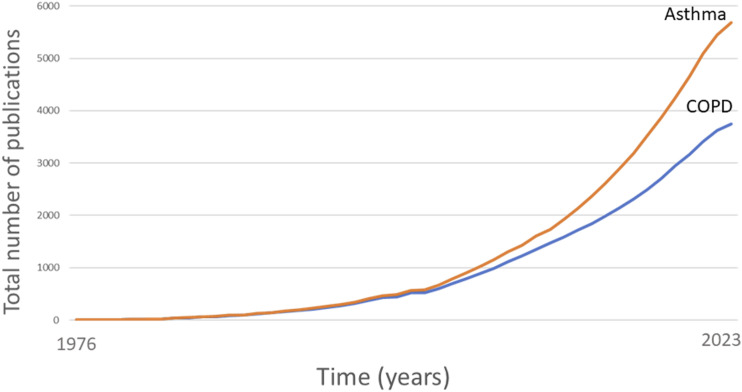
Total number of publications over time that include the terms ‘asthma or COPD’ and self-managment.

Over the last 20 years, evidence around respiratory self-management interventions has accumulated exponentially (Figure 2). The evidence in the asthma self-management field was however already quite convincing in the early 2000s. Gibson et al. (2003)^9^ included 36 studies in their Cochrane review regarding self-management education in the adult asthma population and found significantly reduced hospitalisations, emergency room visits, unscheduled doctor visits, days off work or school, nocturnal asthma, and quality of life. Another Cochrane review that was published around the same time, showed that self-adjustment of medications according to a written action plan gave a similar improvement in health outcomes as adjustment of medications by a doctor.^10^ In 2017, a systematic meta-review of 270 RCTs on asthma self-management confirmed that asthma SMIs reduced unscheduled healthcare use, improved asthma control, and reported that SMIs were applicable to a wide range of target groups and clinical settings, and did not increase health care costs.^5,7^

In contrast, the first small controlled trial aimed at evaluating the effectiveness of a COPD intervention in patients with COPD was published in 1996.^4^ In 2003, the first Cochrane review regarding self-management in COPD was published,^17^ which could only include nine studies, of which eight RCTs. No clear effects were reported.^17^ Two updates of this review, published in 2007^18^ and 2014,^19^ and an extensive individual patient data review published in 2016^20^ all described an improvement of health-related quality of life (HRQoL) and a reduction of hospitalisations in favour of people who received COPD self-management interventions.^6,18,19^ A review evaluating the effectiveness among primary care COPD patients that was published in 2018, did however not report positive effects. Substantial differences between this review and the previous Cochrane reviews, were the population (relatively mild) and limited use of action plans (33%). In 2022, another Cochrane update was published and reported, in line with the previous updates, improvements in HRQoL and a lower probability of respiratory-related hospital admissions.^6^ Evidence therefore seems to suggest that COPD SMIs have positive effects on group level.^6,21–23^

As measuring solely patient’s health outcomes, self-efficacy, and HRQoL may not fully reflect the effects of behavioural interventions like SMIs,^24,25^ intermediate outcomes such as motivation, activation, self-management skills, and actual health behaviours are now measured more frequently in recent SMI studies.^6,11^ Performing meta-analyses with these outcomes is however still challenging because of low study numbers and heterogeneity in outcome measures.

### Intervention characteristics

In 2003, Gibson et al.^9^ identified that clinically important improvements in asthma health outcomes were most evident in interventions involving: (1) education; (2) a written action plan; (3) self-monitoring; and (4) regular medical review. After an unsuccessful attempt of Deford et al.,^26^ 10 years later, to validate these data, it was suggested that other content could be equally important. A review of Pinnock et al. in 2017,^5^ including 270 RCTs, reported that (1) education, (2) a written action plan, and (3) the support of regular professional review were the core components of effective asthma SMIs.

A meta-analysis of individual patient data found that COPD SMIs running over a longer period did better.^22^ This is not surprising as it will take time to build up experience and receive feedback on problem solving and decision-making behaviour.^22^ The factor ‘intervention duration’ could however not be confirmed as an effective component by sub-analyses performed in the most recent COPD self-management Cochrane review.^6^

### Patient characteristics

Whereas there are many patient characteristics that can potentially influence outcomes of SMIs (e.g. health literacy, comorbidities, cultural background, socio-economic status, support), there is no evidence for certain subgroups of respiratory patients benefitting most from the intervention.^6,20^ Using individual trials to identify patient subgroups that benefit most from respiratory SMIs is complicated, as these trials usually lack power.^27^ In 2016, a meta-analysis of individual patient data pooled 3282 COPD patients included in 14 trials.^20,22^ Whereas some subgroups did better for certain outcomes, none of the subgroups showed a consistent effect on all relevant outcomes.^20^ The authors therefore concluded that limiting SMIs to specific COPD patient subgroups could not be recommended.^20^

### Concerns regarding harmful effects

In 2012, Fan et al reported significantly higher mortality rates in the SMI group of a large RCT including severe COPD patients.^28^ The trial was stopped prematurely and sparked concerns about the safety of COPD SMIs and it was recommended to use data monitoring committees in future behavioural intervention studies.^28^ Available data could not fully explain the excess mortality^28^ but the fact that COPD-specific action plans were used in a severe COPD population with frequently existing comorbidities may have played a part.

In 2017, a Cochrane review regarding the effects of COPD SMIs, including exacerbation action plans, found a very small, but higher respiratory-related mortality rate in the self-management intervention group compared to the usual care group.^21^ One of the studies dominating this negative effect was not surprisingly the Fan study.^21,28^ In the 2023 Cochrane review on COPD SMIs, more studies could be included in mortality analyses and no excess on respiratory-related and all-cause mortality risks was reported.^6^ This strengthens the view that COPD SMIs are unlikely to cause harm.

Some asthma SMI studies have reported negative intervention impacts among children, such as increased levels of emergency departments, this may however reflect the content of self-management information delivered to children including recommendations as e.g. to contact a health care professional (HCP) in case of an exacerbation.^11^

## Action plans

Two decades ago, it was already concluded that asthma interventions without a written action plan were less efficacious^9^ and that the efficacy of self-management education was similar regardless of whether patients self-adjusted their medications according to an individual written plan or whether the medication adjustments were made by a doctor.^10^ The current asthma guidelines state that all patients with asthma should be provided with a written action plan, in addition to asthma education and regular review by a HCP.^7^

In COPD, a 2017 Cochrane review comparing SMIs including exacerbation action plans with usual care concluded that SMIs that include COPD exacerbation action plans are associated with improvements in HRQoL and a lower probability of respiratory-related hospital admissions.^21^ Generating evidence comparing SMIs with and without an action plan is difficult, as the majority of COPD SMIs include exacerbation action plans.^6^ Written exacerbation action plans are nowadays recommended as an integral part of COPD management by COPD guidelines.^8^

Benefits from the use of action plans arise from earlier initiation of appropriate treatments, resulting in less severe exacerbations, accelerated recovery, and reduced healthcare utilisation.^7,29,30^ The actions should be discussed, understood, agreed, and reinforced with the patient regularly.^31^ COPD exacerbation action plans specifically promote self-management of exacerbations via the patient’s early recognition of the development of an exacerbation.^32^ Defined actions may vary from contacting an HCP to starting self-treatment with oral corticosteroids (and antibiotics).^21^

Action plans should always be offered with a decent training in the use and with provision of feedback over time to improve patients’ self-management skills.^25,31^ There is no evidence for using an asthma or COPD exacerbation action plan if not combined with support of an HCP.^29,31^ A meta-analysis on hospitalisations in a Cochrane review regarding ‘brief patient education interventions for exacerbations in COPD’ showed some positive effects but only included two studies.^29^ The study that dominated the analysis (92.3% of the weight) included a formal patient training program and case-manager support and could clearly be classified as a SMI.^33^

For patients with COPD it is important to maintain adequate exercise and diet, refrain from smoking, and look after their wellbeing and mental health, support for changing health behaviours is therefore crucial.^34^ Depending on a patient’s needs, action plans for e.g. physical exercise, nutrition, stress-management and breathlessness should be considered. All action plans may be printed, digital, or pictorial, to suit the patient’s needs and literacy.^7^

## Patient’s adherence

When people do not adhere to treatment, no treatment successes can be expected, adhering to treatment is therefore crucial.^35^ Patients’ adherence to SMIs can be influenced by structural, disease-related, social, and psychological factors.^36^ Estimated adherence rates with inhaled corticosteroids vary between 30% and 70% in asthma patients.^37^ Non-adherence to asthma treatment is associated with poor symptom asthma control, reductions in HRQoL and higher health-care use.^38^ It has been reported that COPD patients are only adherent to approximately half of their therapies and poor adherence is associated with increased exacerbations, hospitalisations, and diminished HRQoL, as well as increased mortality and economic burden.^35,39^ Adherence rates to COPD SMIs lie between 42% and 60%.^40–42^

Patients may be more likely to seek health-improving behaviours in order to prevent their condition or reduce its progression when they are able to perceive the risks associated with a given condition.^43,44^ Being aware of the benefits of treatment, however, does not guarantee motivation or willingness to be adherent.^35^ A person’s willingness, motivation, and ability to engage in SMIs may vary depending on factors such as ethnicity, literacy, understanding of health concepts, numeracy, beliefs about the disease and medication, desire for autonomy, and the health care system.^7^ To increase the patient’s adherence to SMIs, interventions should be tailored to individual’s needs, health beliefs, and capabilities as each individual may need a different approach to tackle intentional and unintentional barriers affecting adherence.^25,34,45,46^ Anticipating individual barriers and facilitators is not only expected to increase adherence, but also to stimulate patients’ intrinsic motivation to change behaviour, optimise self-management skills, and therefore improve outcomes.^46^

## The use of theories

A recent review reported that having a theory-based SMI, added positively to implementation.^11^ Underpinning theories have been used infrequently for SMIs over the last two decades. A systematic review regarding self-management strategies in primary health care practice reported that only 28.1% of the interventions were underpinned by a theoretical framework.^47^ Theoretical frameworks can inform the content of an intervention in a more holistic manner and/or they can lead to the choice of certain pedagogical techniques and/or delivery style.^11^ Details regarding the distinctive way theories have been used are important, but often not provided.^11^ Some of the theories previously used in SMIs are listed in Text Box 1.

**Text Box 1.** Some underlying theories/models previously included in self-management interventions.

**Bandura’s social cognitive theory^97,98^**

The theory holds that behaviour is determined by expectancies and incentives. E.g. patients who value the perceived effects of changed lifestyles (incentives) will attempt to change if they believe that (1) their current lifestyles pose threats to any (health) outcomes; (2) that particular behavioural changes will improve (health) outcomes; and (3) that they are personally capable of changing their behaviour.

**Health belief model (HBM)^44,98^**

The HBM is based on the premise that people's beliefs and perceptions about health risks and the benefits of taking action, influence their health-related decisions and behaviours. The HBM hypothesizes that health-related action depends upon the simultaneous occurrence of three classes of factors: (1) the existence of motivation or health concern; (2) the belief that there is a threat or serious health problem; (3) the belief that a particular health recommendation would be beneficial in reducing the perceived threat or health problem at an acceptable ‘cost’ (perceived barrier).

**Transtheoretical model (=stages of change model)^99^**

This model hypothesizes that individuals move through stages of change: pre-contemplation (unaware of problem), contemplation (aware of problem and of the desired behavioural change), preparation (intends to take action), action (practices the desired behaviour), and maintenance (works to sustain the behavioural change). When a patient is in the early stages of change, action-oriented guidance is likely less efficient, a personalised education approach could facilitate moving patient readiness toward the 'preparation' or 'action' stage.

**Cognitive behavioural therapy (CBT)^100^**

Traditional CBT concentrates on how an individual’s thoughts, behaviours, and emotions are connected.^101^ CBT is a combination of Behaviour Therapy and Cognitive Therapy, which focuses on the ‘here and now’, and is based on the premise that cognitions influence feelings and behaviours and that subsequent feeling and behaviours can then influence cognitions.^102^ The basic principles of CBT can be summarised as: (1) it is interpretations of events, not events themselves which are crucial; (2) what we do has a powerful influence on our thoughts and emotions; (3) mental-health problems are best conceptualised as exaggerations of normal processes; (4) it is usually more fruitful to focus on current processes rather than the past; (5) it is helpful to look at problems as interactions between thoughts, emotions, behaviour and physiology and the environment in which the person operates; (6) it is important to evaluate both our theories and our therapy empirically.^100^

## Tailoring of patient-centred approaches

The components and behaviours that SMIs target haven’t significantly changed over the last 20 years (Table 2). However, whereas two decades ago the SMIs were more set up as ‘one size fit all’, using rigid non-personalised intervention structures, the importance of taking into account the patient’s needs, preferences, and capabilities is now recognised.^8^ Shared decision-making will place the patient’s needs and perceptions central in the communication between patients and HCP and empower and motivate the patient.^46^ Within SMIs, patient-centred interactions should focus on: (1) identifying needs, health beliefs and enhancing intrinsic motivations; (2) eliciting personalised goals; (3) formulating appropriate strategies (e.g. exacerbation management) to achieve these goals; and (4) evaluating and re-adjusting strategies.^14^

**Table 2.** Frequently used COPD self-management intervention components, potential content, and related health behaviours.^a^

Component	Potential content	Health behaviour
Disease knowledge^b^	- Education	Preventive measures
	- Decision making	Medication adherence
		Lifestyle modifications
Smoking cessation	- Education	Live in a smoke-free environment
	- Smoking cessation intervention	
	- Decision making - action plan	
Medication adherence^b^	- Education	Medication adherence
	- Skills training (effective use inhaler)	
	- Decision making - action plan	
Exacerbation prevention / management^b^	- Education	Prevent and seek early treatment of COPD exacerbations (e.g. action plan use and vaccinations)
	- Skills training (self-monitoring, training to use the action plan)	
	- Decision making - action plan	
Physical activity	- Education	Maintain an active lifestyle
	- (Home) exercise program	
	- Decision making - action plan	
Breathlessness management	- Education	Maintain comfortable breathing
	- Skills training (breathing exercises)	
	- Decision making - action plan	
Stress management	- Education	Manage stress and anxiety
	- Skills training (relaxation and breathing techniques)	
	- Decision making - action plan	
End-of-life management	- End-of-life conversations	Advanced care planning
	- Advance directives	
Energy conservation	- Education	Conserve energy
	- Decision making - action plan	
Diet	- Education	Keep a healthy diet
	- Decision making - action plan	
Avoiding triggers^b^	- Education	Avoiding aggravating factors (e.g. smoke, pollution)
	- Decision making - action plan	

Abbreviations: COPD: chronic obstructive pulmonary disease.

^a^Information included from the following publications.^6,8,48,49^

^b^Components frequently included in asthmas studies + medical review.^5,50^

### Partnership of patient and health care professional

The term ‘self’ implies self-care and yet self-management always includes a partnership with an HCP.^13^ In 2002, Bodenheimer et al. already described the importance of having a patient-HCP partnership, with HCPs having a positive attitude towards self-management and thus recognising patients as experts on their own disease.^51^ The importance of a patient-centred partnership is now, 20 years later, clearly recognised in the Asthma guidelines, with the importance of the patient-HCP communication discussed and emphasised.^7^ This is different in the COPD field, with e.g. the GOLD guidelines focusing on defining the HCP’s coaching role instead: ‘strategies, techniques and skills used by HCPs to arm patients with the knowledge, confidence and skills required to self-manage their disease effectively’.^8^ The patient-HCP partnership or the importance of communication are not discussed.^8^

The HCP should ideally serve as the patients’ companion within the complicated world of health care and support the patient in gaining knowledge, skills, tools, and confidence to become more active in their own care and positively change their behaviour.^34,52^ The initial communication should focus on identifying needs, health beliefs, enhancing intrinsic motivations, eliciting personalized goals, and formulating appropriate strategies to achieve these goals (ref Effing 2016).^14^ HCPs need to go beyond pure education/advice-giving (didactic) approaches.^8,24^ A continuous process should be applied to provide problem-solving support and to evaluate and reinforce positive changes in patients’ comprehension, attitudes, skills, confidence, motivation, and behaviours, and to determine if the pre-established goals and objectives are met.^48^ A positive attitude toward SMIs, sufficient time, and training are requirements for HCPs to be involved in SMIs.^53^ Factors that may negatively affect the patient-HCP communication, include inconsistent messages from different HCPs, limited consultation time, use of technical language, failure to account for cultural differences, and reduced health literacy, especially as it relates to written communication.^54^ HCPs should be able to understand and place in context patients’ ideas, concerns, and expectations, as well as their physical, social, and psychological situation.^16^

The required self-management training for the HCPs is still not clearly defined,^24^ but a course in behavioural change techniques as, for example, motivational interviewing, is highly recommended as a minimum education.^25^ The HCP should work in close collaboration with other HCPs and have access to medical data,^13^ so communication around the patient can be facilitated and inter-disciplinary alignment ensured.^48^ In the asthma population, the delivery of SMIs by long term conditions educators, community health workers, or peer counsellors may also lead to improved outcomes.^5,55,56^

SMIs in respiratory patients may involve varying degrees of independence,^7^ depending on factors such as disease severity, co-morbidities, and access to health care.^13^ In all asthma patients, regardless of the disease severity, SMIs that included scheduled follow-up with HCPs of totalling at least 2 hours were more effective in reducing healthcare use and improving quality of life.^31^ The amount of HCP support is a major driver of costs.^31^ In asthma, the time investment in supporting people to develop self-management skills is offset by time saved in providing acute care.^31^ In COPD self-management, there is also some positive evidence around costs-effectiveness.^57,58^ Generating more cost-effectiveness data around SMIs will be important for implementation purposes. It is also essential that HCPs deliver interventions as outlined in protocols. The quality and quantity of fidelity reporting of intervention deliveries is still incredibly low.^59^

### Tailoring of interventions

In an ideal world, SMIs should be completely tailored to the patient’s needs, preferences, and capabilities to elicit the patient’s motivation and adherence.^25,34^ If possible, factors such as (digital) health literacy, readiness level, exacerbation frequency, comorbidities, support level, cultural background, and developmental stage (in case of children) should be considered while tailoring SMIs.^7,24,25^ Lack of HCP’s time is however already a significant barrier for implementing current standardised SMIs^60^ and further tailoring of SMIs in practice will likely costs more HCP time investment. Developments in digital technology may be able to support efficient tailoring of SMIs in the future.^61^

#### Health literacy

Health literacy can be defined as a person’s ability to access, understand, evaluate, communicate, and use health-related information.^62^ It is increasingly recognised that the patient-HCP partnership and the patient’s engagement can be hindered by the patient’s health literacy.^63^ Health literacy is also known to be negatively associated with patients’ self-efficacy and self-care behaviours, affecting individual decisions, actions, and lifestyle behaviours.^64–66^

Health literacy is not only determined by patient-related influences (e.g. cognitive abilities or literacy skills), but also by HCP-related influences (including communication skills), as well as health system provision of easy access to needed information and services.^54^ To adapt for patient’s health literacy and to optimise patient-HCP communications, changes can be made on both the intervention’s content and delivery modality: e.g. using pictorial aids,^67^ simplifying ‘one-step’ action plans,^25^ avoiding medical jargon,^68^ creating informed care providers,^69^ and adopting telecommunication and virtual care services.^69^ There is potential to improve health literacy among higher-risk populations, but research regarding this remains underdeveloped and effects on health inequity are still largely untested.^70^

#### Readiness level of a patient

Only a minority of patients are prepared to immediately change their health behaviours.^71^ When the HCP assumes that the patient has greater readiness to change their behaviour than there actually is, resistance and patient non-adherence will be likely.^72^ Checking the patient’s current level of readiness and integrating features in the self-management intervention to optimise the patient’s willingness to change their behaviour is advisable.^25^

#### Exacerbation frequency

In COPD, the use of exacerbation action plans in people with very low exacerbation rates seems impractical as they may have forgotten all about their exacerbation action plans once they have an exacerbation. Targeting those who have a higher exacerbation rate with exacerbation action plans, may be a better allocation of resources.^21^

#### Comorbidities

Multimorbidity has been reported in up to 70% of the elderly population across several low-, middle- and high-income countries.^73^ Specifically, in the (older) population of people with COPD, comorbidities are common.^74^ Comorbid physical, mental, and/or cognitive dysfunction may conflict or confuse disease specific self-management approaches and prevent patients to successfully self-manage.^34,47^ Exacerbation action plans solely directed towards the respiratory disease, may be unsafe as comorbid symptoms may overlap with respiratory symptoms and potentially lead to incorrect actions and delay in proper treatment.^25^ Respiratory exacerbations may also trigger deterioration of comorbid conditions (e.g. increases of blood glucose levels because of prednisolone treatment; increase of anxiety levels because of dyspnoea).^25^ Self-management approaches used in people with multiple diseases, should therefore be individually tailored.^25,34^ Their management may also benefit from a multidisciplinary team-based approach,^75^ in which HCPs of different disciplines work together.^74^

#### Social support

Positive social support may be associated with improved health outcomes in COPD patients^76^ and better disease management behaviours.^77^ Involvement of family and peer support in SMIs has increased over the last two decades^6^ and has the potential to improve communication and facilitate the development of empathic patient-HCP relationships within SMIs.^78^

#### Cultural factors

Globally, migrants living outside their country of birth make up a substantial proportion of the chronic respiratory disease population.^79^ Language, cultural differences, religion, and socioeconomic status may complicate access to interventions, interaction with HCPs, and intervention adherence.^79,80^ Cultural norms may influence the patient’s empowerment to act as an informed decision-makers, their partnerships with HCPs, their disease self-management, and their access and navigation of health information.^81,82^ To date, strategies to implement culturally and linguistically responsive interventions have been developed but appear to be poorly applied.^79^

#### Paediatric patients

The school can be an important facilitator for implementation of asthma self-management interventions for children and adolescents.^50^ Communication needs to be adapted to the child’s developmental stage and directed to the child itself.^50^ Digital applications and health interventions have the potential to promote their engagement.^50^ During adolescence, the responsibility for own medication intake increases which can negatively affect medication adherence.^83^ Self-management behaviour could be increased by focusing on areas in which the adolescent is not confident using an empathic approach to identify beliefs and behaviours that may prevent optimal treatment (e.g. impact of treatment on their physical or sexual capabilities).^7^ Finally, strategies should be tailored to the patient’s stage of psychosocial development and desire for autonomy; adolescents are often focused on short-term rather than long-term outcomes.^7^

### Incorporation of digital technology

Technology has become a normative part of life and the use of digital applications within SMIs is therefore a natural step. Due to the COVID-19 pandemic, health organisations have started to rapidly scale up, synergise and expand digital health innovations.^84^ The use of digital technology may assist with HCPs contacts, especially when direct accessibility to HCPs is limited because of e.g., distance.^85^ Digital health apps can also complement or replace paper-based self-management plans, with advantages of improved accessibility, providing prompts if symptoms deteriorate, and abilities to further tailor materials to the patient’s needs, comorbidities, health literacy etc.^61^ Digital apps can incorporate educational tools, provide feedback to the patient, include tools to avoid exacerbation triggers, include inhaler technique training, check inhaler medication adherence, and support capturing and collating patient data.^61,86^

Interventions that use elements of both face-to-face and internet-based interventions are often referred to as ‘blended interventions’.^87^ A review regarding blended SMIs, including seven asthma and 15 COPD RCTs, reported beneficial effects but had to conclude that due to limited studies included in meta-analyses, findings had to be interpreted cautiously and that more research was needed.^85^

There are currently many asthma and COPD apps and platforms available that incorporate tools for e.g. self-management and symptom tracking, of which some have even been adopted by public health bodies.^88^ A recent review of the current evidence regarding such an app, ‘myCOPD’, concluded that whereas the app is promising for self-managing COPD, the clinical benefit is still uncertain because of limitations in the evidence.^89^

Knowing that education or self-monitoring as an isolated intervention have been proven to be ineffective,^29,31^ providing apps that solely offer this should be used with extreme care. Whereas the use of digital technology may be a facilitator for some (e.g. adolescents, people interested in technology), it may be a barrier for others (e.g. lower sense of technological self-efficacy, difficulties to interpret and act upon device readings).^90^ When incorporating digital technology into existing SMIs, important considerations will be to: (1) tailor to the patient’s digital literacy and skills, (2) ensure accessibility and inclusivity, (3) optimise patient and HCP digital engagement and perceived usefulness, (4) ensure that digital healthcare solutions are safe to use, and (5) avoid undue self-management load because of intensive monitoring.^61,90^

Incorporation of effective and engaging digital health innovations into SMIs has great potential, some examples are (1) the use of artificial intelligence (AI) and machine learning approaches to predict exacerbations in action plans; and (2) the continuous tailoring of SMI materials based on e.g. disease activity and progress.^61,86^ As with SMIs in general, any digital application should be designed with the end user in mind. However, it is also important to facilitate uptake by HCPs to allow for successful integration into existing healthcare systems.^86^ Finally, tools and security measures are necessary to manage the large amounts of data that will be generated.^86^ Building up evidence around digital health innovations to ensure evidence-based practice is of utmost importance.

### Incorporation of behavioural change techniques (BCTs)

In 1994, Clark et al. already reported that self-management programs must be based on a sound theoretical understanding of behaviour change to be successful and employ self-management strategies designed to improve knowledge, skills, and feelings of self-control.^91^ More recently, several systematic reviews have included BCT taxonomies to try to identify techniques associated with increased effectiveness in complex behavioural interventions.^6,23,26^

BCTs are defined as ‘an observable, replicable, and irreducible component of an intervention designed to alter or redirect causal processes that regulate behaviour’.^92^ BCTs can be used alone or in combination, and in a variety of intervention forms (e.g. face-to-face, written or digital).^92^ Within SMIs BCTs are used to elicit the motivation, confidence, and competence of participants.^14^

According to guidelines, optimal asthma SMIs should include ‘education’, ‘self-monitoring of asthma symptoms’, use of a written action plan’, and ‘regular review’.^7^ Depending on their delivery, these four components can be linked to multiple BCTs.^26,92^ It is unclear which combination of BCTs leads to optimal effectiveness.^26^ A meta-regression analyses of asthma ‘self-care’ interventions found that ‘active involvement of participants’ was associated with a reduction in unscheduled health care use.^26^

BCT clusters that are most frequently integrated in COPD SMIs to promote the uptake and optimal self-management behaviour are ‘goals and planning’, ‘feedback and monitoring’, ‘shaping knowledge’, and ‘social support’.^6^ None of the current COPD SMI have however found any associations between the number of included BCTs and improvement in HRQoL nor respiratory-related hospitalisations^6,23^ and there is no evidence regarding the combination of BCTs that should be incorporated in interventions to maximise its effectiveness. A review reported that relatively few COPD SMIs targeted mental health, which they found surprising as the highest effect sizes were found in those studies that utilised BCTs to target mental health concerns.^23^ It is however possible that the type of BCTs rather than the targeted behaviour influences the intervention effect.^23^ More research on BCTs is needed.^6,23^

## Self-management interventions implementation

In 2014, a national review of asthma deaths in the United Kingdom, reported that in only 23% of the people who died, documentation could be found that self-management education had been provided and only 45% of people who died had sought or received medical attention in their final attack.^93^ The implementation of SMIs in health systems, is still sub-optimal.^94^ Barriers to adopting SMIs in organisations are time, the need to develop professional skills, and tackling negative views about the usefulness of intervention.^31^ A recent review regarding school-based asthma SMIs concluded that no single condition appeared in isolation as a trigger for successful implementation of an intervention, but that theory-based intervention, having good levels of engagement from parents, positive experiences among children, and involvement of school nurses contributed to successful implementation.^11^

In both COPD and asthma, it has been reported that for effective implementation of SMIs, a whole systems approach is required in which active engagement of patients is combined with the training and motivation of HCPs working in organisations that value SMIs.^20,95^ Ideally, health care systems should be able to: (1) support SMIs; (2) address the multi-morbidities with which the patient is living; (3) provide flexible access to professional advice; and (4) ensure continuity of care.^34^ Implementation research is necessary to direct the implementation of respiratory SMIs on a larger scale, with policy, funding, and cost-effectiveness being most likely important determinants.^16^

SMI frameworks aim to guide system-wide changes in service delivery for chronic disease management.^96^ Only eight frameworks for provision of SMIs were identified by a recent review, of which five focussed on respiratory conditions.^96^ There is little evidence of active engagement between policy makers in different countries to learn from each other.^96^

## Conclusion

In the last 20 years, the body of evidence around self-management in both asthma and COPD has grown exponentially and SMIs are now recognised in asthma and COPD guidelines (Table 3). Whereas over this whole period, developments in the asthma self-management field have been ahead of COPD, future challenges seem to be similar in both fields. Whereas asthma and COPD SMIs lead to better outcomes on group level, the patient’s adherence to the interventions and the implementation of SMIs are not satisfactory. Recommendations have been listed in Text Box 2.

**Table 3.** Achievements in the field of respiratory self-management over the last two decades.

Asthma	COPD
Evidence around the effectivity of SMIs	Evidence around the effectivity of SMIs
Recognition of asthma self-management in guidelines	Recognition of COPD SMIs in guidelines
Definition of core components of SMI	Conceptional definition of a COPD SMI
Recognition of the importance of patient-HCP partnership and communication in guidelines	Recognition of the importance of HCP coaching in the guidelines
Implementation in practice	Implementation in practice

Abbreviations: COPD: Cchronic obstructive pulmonary disease; SMI: self-management intervention; HCP health care professional

**Text Box 2.** Recommendations for the future.

- Promote the use of theories during SMI development to underpin its content and delivery methods and increase effectivity and implementation
- Develop HCP’s training around shared decision making, patient-HCP communication, the use of behavioural change techniques, and individual tailoring of SMIs
- Provide SMI interventions that include shared decision making and can be tailored to the patient’s needs, health beliefs, and capacity to improve patient’s motivation, adherence, and outcomes
- Build up evidence around the use of digital applications integrated in SMIs
- Perform implementation research to create knowledge about how to best facilitate implementation of SMIs on a larger scale

Abbreviations: SMI: self-management intervention; HCP health care professional.

Patient’s motivation and adherence could most likely be improved by using more patient-centred and individually tailored SMIs, considering the patient’s needs, health beliefs, and capacity (e.g. (digital) health literacy, readiness level, comorbidities, social support, and cultural background). Incorporation of digital technologies should be considered to improve the quality, efficiency, and/or accessibility of interventions. However, research will be of utmost important to ensure evidence-based practice. In addition, patient-HCP communication needs to be optimised, this is not only important for the field of respiratory SMIs, but also for the larger medical profession. Education and training in patient-centred communication should ideally get more weight in professional training and the respiratory self-management community should develop (accredited) training courses around SMIs including training in the use of patient-centred shared decision making, behavioural change techniques, goal setting, and evaluations. Having underpinning theories for the SMI’s content, techniques, and delivery styles may positively influence implementation (and effectivity) of the intervention. Finally, implementation research is necessary to evaluate how the implementation of SMIs on larger scale can be facilitated. Cost-effectiveness, policy (makers), and funding alignment will be major determinants for this.
